# Perceptions of HIV infected patients on the use of cell phone as a tool to support their antiretroviral adherence; a cross-sectional study in a large referral hospital in Kenya

**DOI:** 10.1186/1471-2458-13-987

**Published:** 2013-10-21

**Authors:** Florence Kinyua, Michael Kiptoo, Gideon Kikuvi, Joseph Mutai, Adrienne FA Meyers, Peter Muiruri, Elijah Songok

**Affiliations:** 1 Kenya Medical Research Institute, Nairobi, Kenya; 2 Institute of Tropical Medicine and Infectious Disease, Jomo Kenyatta University of Agriculture and Technology, Nairobi, Kenya; 3 Department of Medical Microbiology, University of Manitoba, Winnipeg, Canada; 4 National Laboratory for HIV Immunology, Public Health Agency of Canada, Winnipeg, Canada; 5 Kenyatta National Hospital, Nairobi, Kenya

**Keywords:** HIV, ART Adherence, Cell phone

## Abstract

**Background:**

Clinical trials were conducted to assess the feasibility of using a cell phone text messaging-based system to follow up Human Immunodeficiency Virus (HIV) infected patients on antiretroviral (ARTs) and assess for improved adherence to their medication. However there is need to evaluate the perceptions of the HIV infected patients towards the use of these cell phones in an effort to better aid in the clinical management of their HIV infection. The objective of this study was therefore to determine the perceptions of HIV infected patients on the use of cell phone text messaging as a tool to support adherence to their ART medication.

**Methods:**

A cross sectional survey was conducted among patients receiving Highly Active Anti-Retroviral Therapy (HAART) at the Kenyatta National Hospital Comprehensive Care Clinic in Nairobi between May and July, 2011. Pre-tested questionnaires were used to collect the socio-demographic and perceptions data. The recruitment of the participants was done using the random probability sampling method and statistical analysis of data performed using Statistical Package for Social Sciences (SPSS) version 16.0.

**Results:**

A total of 500 HIV infected patients (Male-107, Female-307) aged 19-72 years were interviewed. The majority of individuals (99%) had access to cell phones and 99% of the HIV infected patients interviewed supported the idea of cell phone use in management of their HIV infection. A large proportion (46%) claimed that they needed cell phone access for medical advice and guidance on factors that hinder their adherence to medication and only 3% of them needed it as a reminder to take their drugs. The majority (72%) preferred calling the healthcare provider with their own phones for convenience and confidential purposes with only 0.4% preferring to be called or texted by the health care provider. Most (94%), especially the older patients, had no problem with their confidentiality being infringed in the process of the conversation as per the bivariate analysis results.

**Conclusion:**

Cell phone communications are acceptable and in fact preferable over cell phone reminders.

## Background

The World Health Organization (WHO) intensified their advocacy on the provision of antiretroviral therapy (ART) to reduce Acquired Immuno Deficiency Syndrome (AIDS)-related deaths and alleviate fears about HIV in the year 2003 [1]. In embracing this advocacy, Kenya expanded its ART coverage tremendously with percentage of adults receiving ART increasing from 55.3% in the year 2008 to 70.4% in the year 2009 [2]. This consequently intensified the need for more ART programmes in Kenya for successful control and prevention of HIV-related mortality which is affecting the country’s economy by a large margin. In response to this need, the Kenyan government released free ART drugs with the aim of lowering the HIV-related mortality rates. However several studies have shown that drug adherence has remained a challenge even after the provision of free medication [3,4].

Technology innovations in Kenya have been increasing tremendously with the Communication Council of Kenya (CCK) report of 2010 [5] describing a mobile phone penetration rate of 39% with sharp concentrations of 70% found within the urban centers where HIV prevalence has been reported to be high [6]. This prompted more effort into health research to seek the possibility of mobile phones solving the challenge of decreasing adherence to ART medication.

A randomized trial in Kenya by Lester *et al.*[7] assessed the feasibility of cell phone text messaging communication as a tool for improved ARV adherence and reported a 12% increase of self reported adherence among the patients receiving text message reminders as compared to the group receiving the normal standard of care. This favorable outcome on cell phone usage has prompted the need to assess the perceptions of the HIV infected patients in a normal life setting other than the experimental set up. Therefore the main objective of this study was to determine the perceptions of HIV infected patients towards cell phone text messaging as a tool for supporting adherence to ART medication.

## Methods

### Settings

The study was conducted at Kenyatta National Hospital Comprehensive Care Centre (KNH CCC) in Nairobi where approximately 20,000 HIV patients receive their medication with an average daily attendance rate of 200 patients.

### Participants

The study participants were the HIV infected patients aged 19-72 years, already on HAART at KNH CCC in the months of May to July 2011 who were considered having above 90% accessibility to cell phones as a means of communication. Risks and benefits of the study were well explained to each of the participant before they consented in writing. Those who consented to participate in the study were issued with the pre-tested questionnaires to seek information on their basic demographic data, cell phone access and current use and perceptions towards cell phone use as a tool to support adherence. This study was approved by the Kenya Medical Research Institute Scientific Steering Committee and Ethical Review Board (Ref SSC-1935).

### Sampling

The random sampling method was used to choose files of patients attending the CCC. This involved randomly selecting 3 files from a batch of 10 files using randomly generated numbers. Interviews for recruitment of the participants were performed upon exit after they were through with the healthcare provider to avoid interfering with the normal running of the clinic.

### Data collection

Pre-tested questionnaires in English or Kiswahili were used for data collection among the sampled participant.Those that could read and write filled the questionnaires by themselves but those that could not had them filled in an interview format using the language preferred by each participant.

### Statistical analysis

Data was entered using Microsoft Access (Microsoft Corporation, Redmond, Washington) and statistical analysis performed using SPSS version 16.0. We present odds ratios (OR), and 95% Confidence Interval (CI) for factors associated with cell phone access and perceptions towards its use as a tool to support ARV adherence.

Perceptions towards cell phone usage to support ART adherence was determined using the participants response to the following issues: whether they supported the idea, the affordability of cell phone, any fear of their confidentiality being infringed during the communication, whether they had any problem with the language used in the communication, whether they anticipated any hindrance to that communication and lastly whether they anticipated to benefit from that intervention.

The perceptions were then determined from the participants response to the above issues using the following criteria: “Positive perception” was defined as saying ‘Yes’ to issues one, two and six and saying ‘No’ to issues three, four and five, while ‘Negative perception’ was defined as saying ‘No’ to issues one, two and six and saying ‘Yes’ to issues three, four and five. The results were analyzed to give overall percentage of those with positive and negative perceptions.

## Results

### Respondents’ cell phone accessibility and perceptions towards cell phone use as a tool for supporting ART adherence

This study reported almost 99% access to cell phone with majority (77%) of those interviewed using the alarm function on their phones for waking up as compared to only 15% that used it as a reminder to take their medications (Table 1).

**Table 1:** Access and current use of cell phones among the sampled HIV infected patients

**Characters**	**All (%)**	**Male (%)**	**Female (%)**
**Phone ownership (n = 500)**			
Yes	497 (99.4)	193 (100)	304 (99.4)
No	3 (0.6)	0 (0.0)	3 (0.6)
**Send SMS (n = 498)**			
Yes	420 (84.3)	163 (84.9)	257 (84.0)
No	78 (15.7)	29 (15.1)	49 (16.0)
**Alarm use (n = 495)**			
Yes	299 (59.8)	98 (50.8)	201 (65.5)
No	201 (40.2)	95 (49.2)	106 (34.5)
**Alarm purpose (n = 295)**			
Waking up	277 (76.7)	91 (73.4)	186 (78.5)
Reminder to take drugs	53 (14.7)	18 (14.5)	35 (14.8)
Reminder for important events	31 (8.6)	15 (12.1)	16 (6.8)

This study also showed that generally 78% of patients with cell phones had good perceptions towards cell phone usage to support ARV adherence as determined by participant response to the selected questions (Table 2).

**Table 2:** Established perceptions towards cell phone as a tool for supporting ARV adherence

**Characteristic**	**GOOD (%)**	**BAD (%)**
Support the idea	99.2 (YES)	0.8 (NO)
Phone affordability	55.0 (YES)	45.0 (NO)
Confidentiality infringement	92.0 (NO)	8.0 (YES)
Language problem	62.0 (NO)	38.0 (YES)
Anticipation of an hindrance	66.5 (NO)	33.5 (YES)
Anticipation of a benefit	98.4 (YES)	1.6 (NO)
TOTAL	473.1	126.9
Overall perception (%)	**78.9**	**21.1**

**Figure 1:**
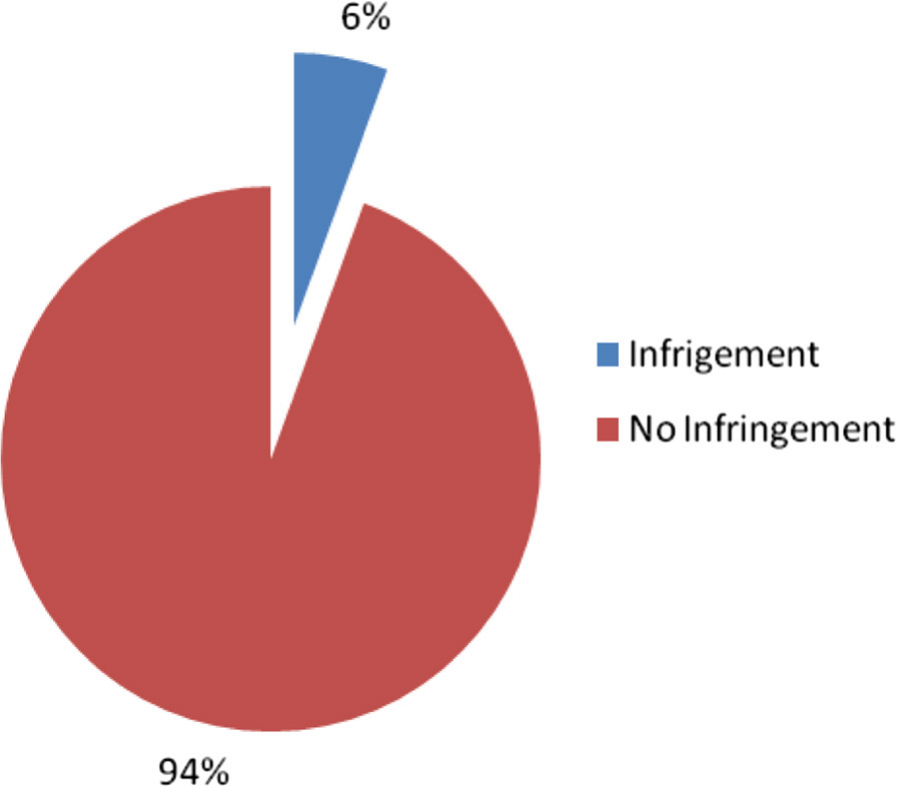
Confidentiality infringement.

Confidentiality infringement was not an issue for 92% of the population although the remainder, mostly the under 30, complained of not being comfortable to share their confidential issues with anyone rather than their family members (Figure 1).

**Figure 2:**
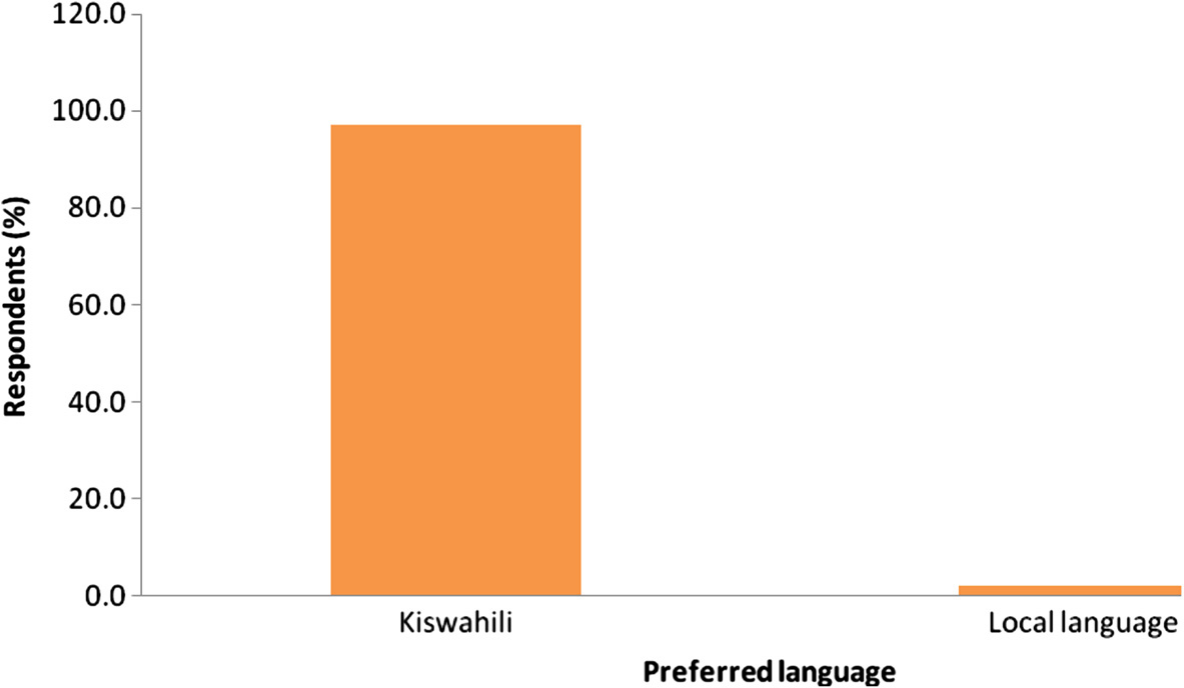
The preferred language for communication.

**Figure 3:**
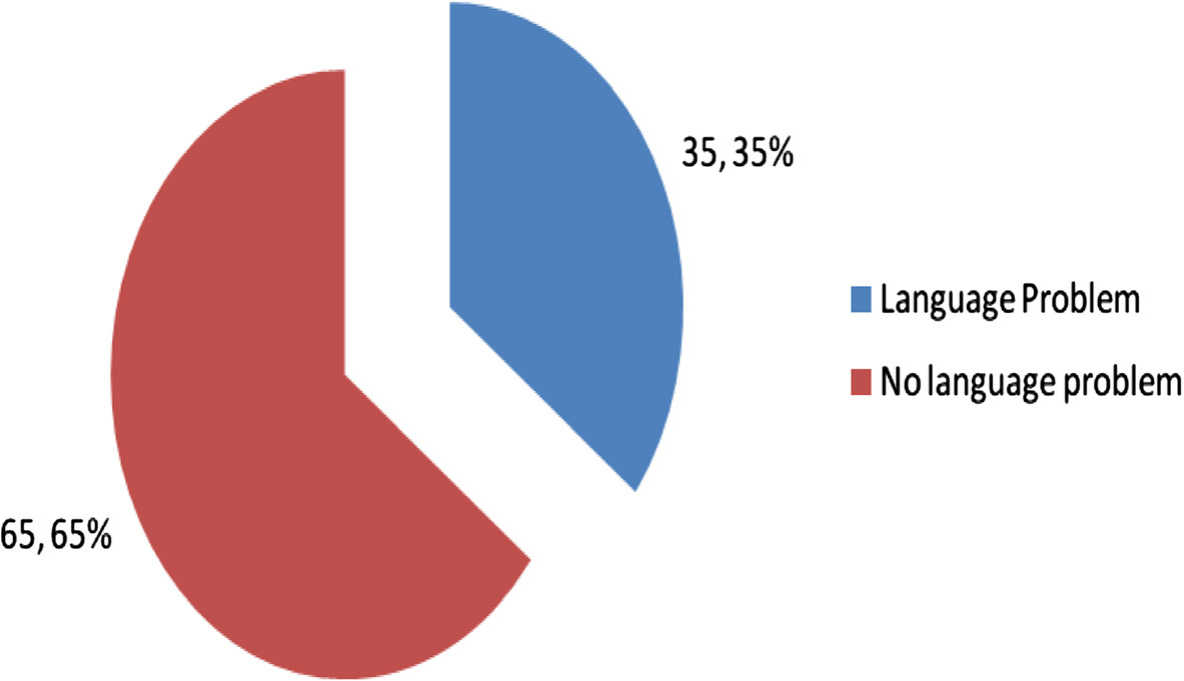
Anticipation of a language problem.

Language concerns (unable to communicate well with English) were an issue among 62% of the population with the majority proposing Kiswahili as the preferred language (Figures 2 &3).

**Figure 4:**
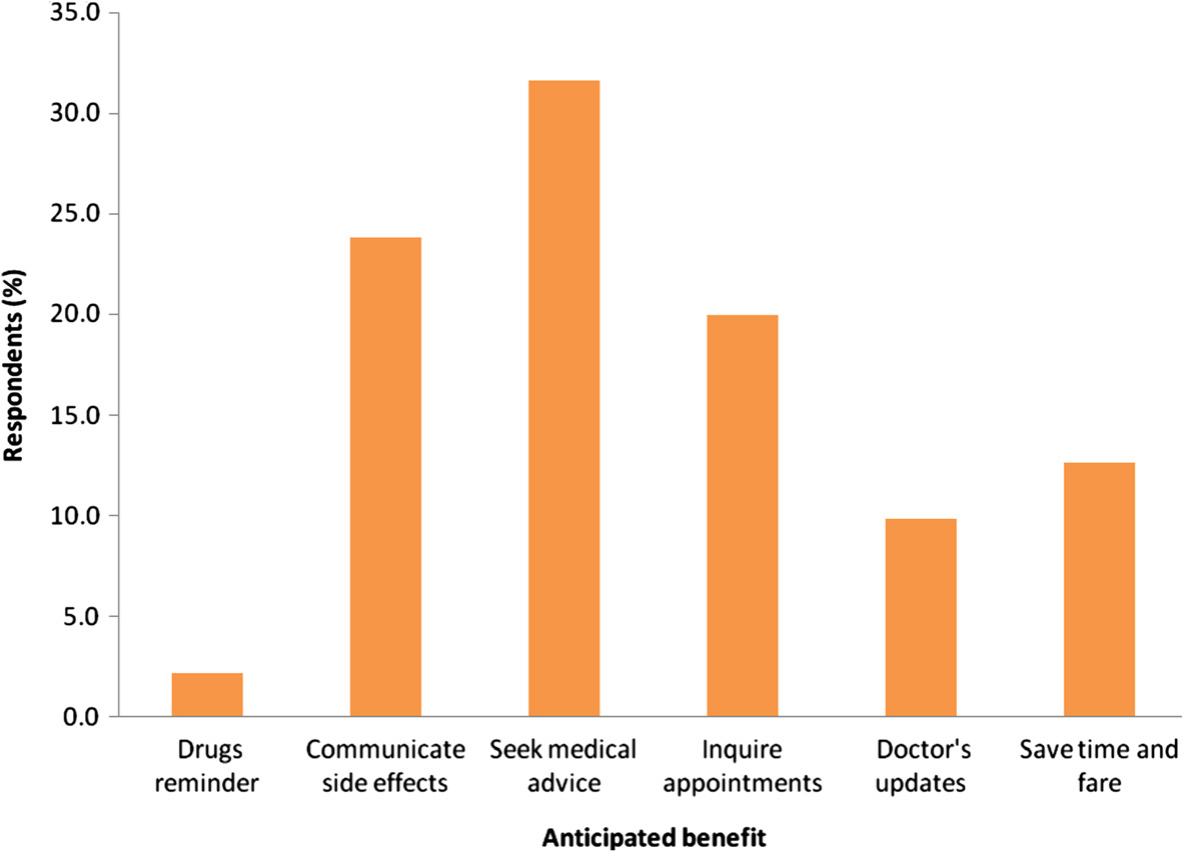
Anticipated benefits from the doctor-patient communication.

A larger proportion (66%) of the population did not anticipate any challenge to hinder the conversation process although rest of the population expressed concern that network problems or language barriers as potential challenges that could interfere with such an intervention in the future. Most (99%) of the population anticipated a benefit from this health care provider-patient communication strategy with the majority claiming to have a chance to receive the required medical advice from the doctor. The majority of these individuals had no problem using their personal cell phones for this communication and they were not ready to share their cell phones in an effort to prevent infringement of their confidentiality (Figure 4).

A large proportion of respondents (72%) preferred only calling the hospital with their own phones for convenience and confidential purposes whereas only 26% could both call or text the hospital and only 0.2% would wait to be called by the hospital. Approximately 47% of the respondents reported that receiving reminders to take their drugs was not the solution for improved adherence and the majority of these preferred face to face communication with the health care provider to seek medical advice (Table 3).

**Table 3:** Participants response towards the mode of communication

**Characters**	**All (%)**	**Male (%)**	**Female (%)**
**Support the idea (n = 499)**			
Yes	495 (99.2)	190 (99.0)	305 (99.3)
No	4 (0.8)	2 (1.0)	2 (0.7)
**Information type (n = 495)**			
Drugs reminder	28 (3.0)	10 (2.7)	18 (3.2)
Communicate side effects	266 (28.4)	106 (28.9)	160 (28.1)
Medical advice	434 (46.4)	171 (46.6)	263 (46.2)
Inquire appointments	183 (19.6)	70 (19.1)	113 (19.9)
Gets healthcare provider’s updates	25 (2.7)	10 (2.7)	15 (2.6)
**Whose phone (n = 499)**			
Own phone	496 (99.4)	192 (99.5)	304 (99.3)
New phone	2 (0.4)	0 (0.0)	2 (0.7)
None	1 (0.2)	1 (0.5)	0 (0.0)
**Communication mode (n = 498)**			
Calling the hospital	359 (72.1)	130 (67.7)	229 (74.8)
Sending SMS to hospital	9 (1.8)	4 (2.1)	5 (1.6)
Calling & texting the hospital	129 (25.9)	58 (30.2)	71 (23.2)
Being called or texted by the hospital	1 (0.2)	0 (0.0)	1 (0.3)
**Phone affordability (n = 499)**			
Yes	261 (52.3)	111 (57.5)	150 (49.0)
No	229 (45.9)	82 (42.5)	147 (48.0)
Don’t know	9 (1.8)	0 (0.0)	9 (1.8)
**Anticipation of a problem (n = 500)**			
Yes	167 (33.4)	66 (34.2)	101 (32.9)
No	333 (66.6)	127 (65.8)	206 (67.1)
**Anticipated problems**			
Affect confidentiality	31 (19.5)	10 (16.7)	21 (21.2)
Network problems	53 (33.3)	26 (43.3)	27 (27.3)
Language problem	52 (32.7)	20 (33.3)	32 (32.3)
Un-cooperative healthcare provider	23 (14.5)	4 (6.7)	19 (19.2)

### Association of different socio-demographic status to the respondents’ opinion

HIV infected patients under 30 years of age were more likely to be careful to protect their confidentiality as compared to those above 30 years of age (p = 0.023) (Table 4). Cell phones were more affordable to the employed and educated as compared to the unemployed and illiterate in the society (p ≤ 0.001) (Table 5).

**Table 4:** Association of the confidentiality infringement with the social demographic characteristics

**Variable**		**Infringement**	**No infringement**	**OR**	** 95% CI**		***p*-value**
**Age**				2.548	1.110	5.848	**0.023**
	≤ 30 yrs	9	74	2.050	1.151	3.652	
	>30 yrs	19	398	0.805	0.622	1.041	
**Gender**				0.513	0.214	1.230	0.129
	Male	7	186	0.634	0.331	1.217	
	Female	21	286	1.238	0.987	1.551	
**Education**				0.957	0.322	2.843	0.937
	Educated	24	402	1.006	0.861	1.176	
	Non Educated	4	70	0.963	0.379	2.448	
**Marital status**				0.617	0.286	1.322	0.216
	Married	12	259	0.781	0.505	1.207	
	Un-married	16	213	1.266	0.905	1.772	
**Occupation**				0.579	0.269	1.244	0.158
	Employed	15	189	1.338	0.931	1.922	
	Un-employed	13	283	0.774	0.517	1.161	

**Table 5:** Association of cell phone affordability with the social demographic characteristics of the respondents

**Variable**		**Afford**	**Not afford**	**OR**	** 95% CI**		***p*-value**
**Age**				0.995	0.621	1.596	0.985
	≤ 30 yrs	44	39	0.996	0.672	1.477	
	>30 yrs	221	195	1.001	0.925	1.083	
**Gender**				1.336	0.929	1.921	0.120
	Male	111	82	1.195	0.955	1.497	
	Female	154	152	0.895	0.719	1.028	
**Education**				0.366	0.217	0.618	**0.001**
	Educated	241	184	1.157	1.071	1.249	
	Non educated	24	50	0.424	0.269	0.667	
**Marital status**				1.343	0.943	1.913	0.102
	Married	153	118	1.145	0.972	1.348	
	Un-married	112	116	0.853	0.704	1.032	
**Occupation**				0.464	0.321	0.669	**0.001**
	Employed	131	73	1.585	1.264	1.986	
	Un-employed	134	161	0.735	0.634	0.851	

Cell phone affordability was not a problem for 55% of the sample population although the rest complained that they were either having no phone of their own (i.e. sharing it with their spouses). The major predisposing factor to this was reported as education (Table 5).

The language problem is more likely to be an issue among the uneducated, unemployed and the aged as compared to the educated, employed and the under 30 years (p ≤ 0.001) (Table 6).

**Table 6:** Association of language problem with the social demographic status of the respondents

**Variable**		**Yes**	**No**	**OR**	** 95% CI**		***p*-value**
**Age**				0.380	0.213	0.678	**0.001**
	≤ 30 yrs	16	67	0.436	0.261	0.728	
	> 30 yrs	161	256	1.148	1.067	1.234	
**Gender**				0.761	0.520	1.114	0.160
	Male	61	132	0.843	0.662	1.074	
	Female	116	191	1.108	0.963	1.275	
**Education**				23.412	10.895	50.311	**0.001**
	Educated	111	315	0.643	0.573	0.721	
	Non Educated	66	8	15.055	7.398	30.637	
**Marital status**				0.812	0.562	1.172	0.266
	Married	90	181	0.907	0.762	1.080	
	Un-married	87	142	1.118	0.921	1.357	
**Occupation**				6.371	4.025	10.085	**0.001**
	Employed	28	176	0.290	0.204	0.414	
	Un-employed	149	147	1.850	1.616	2.118	

Those in need of cell phone reminders were more likely to be 30 years or younger and single versus those above 30 years of age and the married, divorced or the widowed (p ≤ 0.001,0.003) (Table 7).

**Table 7:** Association of perceptions towards cell phone reminder with the social demographic characteristics

**Variable**		**Yes**	**No**	**OR**	** 95% CI**		***p*-value**
**Age**				3.243	1.670	6.299	**0.001**
	≤ 30 yrs	16	66	2.446	1.556	3.844	
	> 30 yrs	29	388	0.754	0.605	0.940	
**Gender**				0.618	0.316	1.210	0.158
	Male	13	180	0.729	0.454	1.168	
	Female	32	274	1.178	0.964	1.440	
**Education**				0.697	0.266	1.830	0.463
	Educated	40	385	1.048	0.939	1.171	
	Non Educated	5	69	0.731	0.311	1.718	
**Marital status**				0.390	0.204	0.745	**0.003**
	Married	15	255	0.593	0.390	0.904	
	Un-married	30	199	1.521	1.207	1.917	
**Occupation**				1.154	0.614	2.168	0.658
	Employed	17	187	0.917	0.621	1.356	
	Un-employed	28	267	1.058	0.832	1.345	

Those using the alarm function on their cell phones were more likely to be above 30 years, females, educated, single and unemployed (p ≤ 0.01) (Table 8).

**Table 8:** Association of perceptions towards alarm use with social demographic characteristics

**Variable**		**Yes**	**No**	**OR**	** 95% CI**		***p*-value**
**Age**				4.421	2.372	8.239	**0.001**
	≤ 30 yrs	70	13	3.620	2.059	6.364	
	> 30 yrs	299	188	0.819	0.762	0.880	
**Gender**				0.544	0.377	0.786	**0.001**
	Male	13	180	0.693	0.557	0.863	
	Female	32	274	1.275	1.094	1.485	
**Education**				0.195	0.113	0.338	**0.001**
	Educated	40	385	1.276	1.167	1.395	
	Non Educated	5	69	0.249	0.154	0.403	
**Marital status**				0.622	0.422	0.894	**0.010**
	Married	15	255	0.809	0.690	0.948	
	Un-married	30	199	1.301	1.058	1.600	
**Occupation**				0.512	0.352	0.744	**0.001**
	Employed	17	187	1.505	1.187	1.907	
	Un-employed	28	267	0.770	0.668	0.887	

## Discussion

The study described here reports the finding that the majority of those using their cell phone alarms used them for waking up rather than reminder for taking drugs which is in agreement with a Peruvian study that reported 77% cell phone accessibility and 23% (7/31) alarm usage as a reminder for their medications [8]. This may imply that non adherence to medication may not be attributed to forgetfulness but other reasons for example side effects, poor feeding or no time for drug refills.

In a similar concurrence with the Peruvian study, most (99%) of those interviewed had no problem using their current phones to call the doctor while the Peruvian study reported 74% of the participants supporting the use of cell phone as tool to support adherence to medication and majority claiming to have no problem using their current phones for the communication.

The anticipation of benefits from the doctor-patient communication than the normal hospital consultation supports other studies [8,9] where most of the respondents reported that they would like to receive general HIV information via cell phones, including advances in HIV treatment and recent research. The low percentage of people with language concerns could be attributed to the growth in literacy level in the country after the introduction of free primary education systems and the fact that most of the people interviewed were from urban settings with increased literacy levels as compared to the rural areas.

However, our findings that majority of the participants would not need the cell phone for reminder to take drugs differed from the Weltel reports [7,10] which indicated that most of those interviewed were willing to use cell phones to receive reminder messages for their HIV medication and that use of text message reminders was a feasible method of improving self reported adherence.

The need for cell phone reminders was high among the under 30 and the widowed concurring with previous study by Huang where younger age and cell phone ownership were significantly associated with acceptance of text message reminders [11].

Confidentiality infringement was not a significant issue among the respondents in the study described here, concurring with Simoes *et al.*[12,13] that in audio computer-assisted self-interview (ACASI), there was more protection of patients’ privacy as compared with the administration of questionnaires by the interviewer.

This also concurred with an Indian study where high proportion (66%) reported using phones to call their healthcare provider to facilitate adherence with loss of privacy not considered a deterrent [14]. The anticipation of benefits from this cell phone communication by a greater proportion has been supported by most of the above studies where populations perceived that HIV information was important to their health. This concurred also with the Atun report on varied health-related uses of SMS applications where he suggested that it “deliver [s] both efficiency savings and improvements in the health of individuals and public health” [15].

The illiteracy reported from the study is also reduced by the enforcement of compulsory free primary and secondary education in the implementation of the new constitution that started in the year 2010. This may also not affect the verbal communication as reported by Curioso *et al.*[8] that the introduction of voice based technology other than the SMS based applications would be of great help to illiteracy in communities.

Similar to other observations, cell phone affordability was not a problem among the educated and employed with a considerable salary implying that an additional income will increase one’s ability to acquire a cell phone.

## Conclusion

Participants generally found the concept of using mobile phones in their health management to be highly favorable. They generally preferred face to face communication with the health care providers versus receiving medication reminders as only a minority currently used their phone alarms for this purpose. They also appeared to favor phone discussions to text messaging communications with health care providers. However although reminders were not preferred or used by the majority, a portion did report using their phone alarms for reminders, so we could not fully conclude that there is no need for reminders, only that they are not a primary reason for use.

## Competing interests

The authors declare that they have no competing interests.

## Authors’ contributions

All authors contributed equally to this manuscript. FK and ES conceived the study; FK conducted the study; MK and GK provided technical assistance; JM assisted in preparation of the data collection tools and AM supported data analysis and write-up, PM assisted in recruitment of the study participants. All authors read and approved the final manuscript.

## Pre-publication history

The pre-publication history for this paper can be accessed here:

http://www.biomedcentral.com/1471-2458/13/987/prepub
